# Neurotensin and Neurotensin Receptors in Stress-related Disorders: Pathophysiology & Novel Drug Targets

**DOI:** 10.2174/1570159X21666230803101629

**Published:** 2023-08-04

**Authors:** Grigorios Kyriatzis, Michel Khrestchatisky, Lotfi Ferhat, Ekaterini Alexiou Chatzaki

**Affiliations:** 1 Laboratory of Pharmacology, Department of Medicine, Democritus University of Thrace, 68100 Alexandroupolis, Greece;; 2 Institute of Neurophysiopathology, INP, CNRS, Aix-Marseille University, 13005 Marseille, France;; 3 Institute of Agri-Food and Life Sciences, University Research Centre, Hellenic Mediterranean University, 71410 Heraklion, Greece

**Keywords:** Neurotensin, stress, anxiety, depression, PTSD, fear memory, ethanol addiction, substance abuse

## Abstract

Neurotensin (NT) is a 13-amino acid neuropeptide widely distributed in the CNS that has been involved in the pathophysiology of many neural and psychiatric disorders. There are three known neurotensin receptors (NTSRs), which mediate multiple actions, and form the neurotensinergic system in conjunction with NT. NTSR1 is the main mediator of NT, displaying effects in both the CNS and the periphery, while NTSR2 is mainly expressed in the brain and NTSR3 has a broader expression pattern. In this review, we bring together up-to-date studies showing an involvement of the neurotensinergic system in different aspects of the stress response and the main stress-related disorders, such as depression and anxiety, post-traumatic stress disorder (PTSD) and its associated symptoms, such as fear memory and maternal separation, ethanol addiction, and substance abuse. Emphasis is put on gene, mRNA, and protein alterations of NT and NTSRs, as well as behavioral and pharmacological studies, leading to evidence-based suggestions on the implicated regulating mechanisms as well as their therapeutic exploitation. Stress responses and anxiety involve mainly NTSR1, but also NTSR2 and NTSR3. NTSR1 and NTSR3 are primarily implicated in depression, while NTSR2 and secondarily NTSR1 in PTSD. NTSR1 is interrelated with substance and drug abuse and NTSR2 with fear memory, while all NTSRs seem to be implicated in ethanol consumption. Some of the actions of NT and NTSRs in these pathological settings may be driven through interactions between NT and corticotrophin releasing factor (CRF) in their regulatory contribution, as well as by NT’s pro-inflammatory mediating actions.

## 1 INTRODUCTION

### 1.1 Neurotensin

Neurotensin (NT), a 1,674 kDa trideca-neuropeptide (pGlu-Leu-Tyr-Glu-Asn-Lys-Pro-Arg-Arg-Pro-Tyr-Ile-Leu-OH) first isolated in 1973, was initially classified as a kinin due to its ability to induce hypotension and vasodilation in rat uterus [1]. Since then, it has been found present and active in the central nervous system (CNS), and in most peripheral tissues. It is expressed in all mammals and is highly conserved between species [2].

NT plays several physiological roles. In the CNS, NT is a neurotransmitter, but it also exerts a role as a neuromodulator of other neurotransmitters, including dopamine (DA) [3], serotonin, acetylcholine, glutamate, and γ-aminobutyric acid (GABA) [4, 5]. NT is stored in vesicles also containing other neurotransmitters, such as DA, suggesting regulatory interactions [6]. In the CNS, it is mainly located in the hippocampus, and also in areas responsible for the control of pain, mediating nociceptive responses and pain relief. NT exerts several actions in the CNS, among others endocrine. These include modulation of pituitary hormones release, control of central blood pressure, inhibition of food intake, and inflammatory processes [7-9]. As a neuromodulator, NT has been involved in the pathophysiology of many neural and psychiatric disorders, more specific schizophrenia [10], eating disorders [11], Parkinson's disease [12], glioma [13], pain [14], cerebral ischemia [15] and epilepsy [2, 4, 5, 9, 16, 17]. Of special interest for neurodegenerative diseases, NT and its analogues can induce generalized hypothermia (between 2-4°C), when introduced directly into the CNS by intracisternal or intracerebroventricular administration [18, 19]. This body temperature reduction has been shown to prevent hippocampal neurodegeneration and preserve locomotor activity during an ischemic insult, suggesting a therapeutic potential for NT [20]. Electrical stimulation of the rat midbrain induces NT release in the prefrontal cortex [6], placing NT and its agonists as adjuncts in the treatment of conditions like schizophrenia [21].

In the periphery, among other actions, NT regulates pro-inflammatory responses and the proliferation of intestinal and cancer cells [22, 23], and has also been implicated in the onset of cardiovascular disease, diabetes, and obesity [24].

Despite its functions, the potential of NT as a therapeutic molecule *via* intravenous (i.v.) administration is somewhat hampered by both its limitation to cross the blood-brain barrier (BBB) and its rapid plasma degradation by endopeptidases and carboxypeptidases. More so, these limits result in a very short half-life (15 min at the central level and 2-6 min in the periphery) [18, 25]. These enzymes act mainly at the NT [8-13] fragment, by hydrolyzing the Arg8-Arg9, Pro10-Tyr11, and Tyr11-Ile12 bonds [26]. As a result, NT functions independently at the central and peripheral levels [22].

## 2 NEUROTENSIN RECEPTORS

The functions and different biological effects of NT are mediated by three distinct receptor subtypes (NTSRs) identified and cloned to date, namely neurotensin receptor 1 (NTSR1), NTSR2 and NTSR3 (Sortilin1). NTSR1 and NTSR2 are G-protein coupled receptors (GPCRs) with seven transmembrane domains (7 TMD) [27]. NTSR3 or gp95/ Sortilin1 is a type I receptor of the Vacuolar protein sorting 10 protein (Vps10p)-containing domain receptor family and is not coupled to a G-protein. This diversity in NTSRs contributes to complex signaling pathways induced by NT and complicates our understanding of NT properties [28].

### 2.1 Neurotensin Receptor 1 (NTSR1)

NTSR1 and NTSR2 belong to the GPCRs superfamily and show high (K_d_ = 0.15-0.5 nM) and low affinity for NT (K_d_ = 5-7 nM), respectively [29, 30]. The NTSR1 gene was first cloned in the colorectal adenocarcinoma cell line, HT-29 [29, 31, 32], and is located on chromosome 20 (20q13), to encode a protein of 424 amino acids (aa) in rats and 418 aa in humans and mice.

NTSR1 is a major mediator of NT. It displays effects in both the CNS and the periphery, including a decrease of blood pressure, analgesia, and hypothermia [33-35]. In addition, it also mediates food intake through paracrine and endocrine signaling in the digestive track [35]. It shows as well oncogenic effects, as it has been implicated in tumor initiation and growth [36]. Interestingly, NTSR1 knock-out (KO) mice are viable and exhibit normal behavior, but present with abnormalities in thermoregulation and motility disorders, showing no hypothermic response after injection of NT, while nociception is not affected [37]. On the other hand, upon experimental loss of function of this receptor, NT interaction with the dopaminergic system is compromised, with resultant disturbances of the mesolimbic DA system by the hormone leptin, inducing hedonic feeding and obesity [38].

NTSR1 signaling leads to the activation of phospholipase C (PLC), which in turn activates the phosphatidyl-inositol-diphosphate pathway leading to the production of inositol triphosphate (IP3) and diacylglycerol from membrane phospholipids [39]. These two second messengers activate protein kinase C (PKC) and induce the mobilization of intracellular Ca^2+^ [40, 41]. Through this signaling pathway, NT can be involved in cell proliferation, survival, migration, and invasion. NTSR1-signaling can be blocked by SR48692, a specific NTSR1 antagonist, or SR142948A, a general antagonist that binds to both NTSR1 and NTSR2 with the same affinity. Moreover, SR142948A can effectively inhibit hyperthermia and analgesia resulting from NT administration in the CNS, suggesting that these functions also depend on NTSR2 when activated by NT [39, 42, 43].

As often in GPCRs, upon agonist stimulation, NTSR1 is internalized and endocytosed [44], further inducing signaling *via* interaction with β-arrestins 1 and 2 [45, 46].

### 2.2 Neurotensin Receptor 2 (NTSR2)

NTSR2 is the second of the NTSRs that was discovered and was cloned from a mouse brain [47]. NTSR2 is a 245 kDa protein of 420 amino acids (aa) in mice and 410 aa in humans and rats. In humans, the gene encoding for NTSR2 is located in chromosome 2 (2p25) [48]. NTSR2, referred to as sensitive to the histamine H1 receptor antagonist levocabastine, is mainly expressed in the brain. Its expression is imprinted in the hippocampus, prefrontal and cerebellar cortex, piriform cortex, olfactory bulb, stria terminalis, preoptic nucleus, amygdala, thalamic nucleus, substantia nigra, ventral tegmental area and hypothalamus, as well as other CNS sites [47, 49-52]. Besides neurons, NTSR2 is expressed in other regulatory neural cells, such as glial cells, mainly astrocytes, as we have recently shown [53], and ependymal cells [54-56].

NTSR2 is involved in NT-induced analgesia but also in response to brain lesions. Indeed, the expression of *NTSR2* mRNA is altered in glial cells in mouse models of brain damage [57]. Also, we have shown that astroglial NTSR2 is implicated in neuroinflammation occurring post-epilepsy *in vivo* and in induced inflammation *in vitro* [53].

The signaling pathway triggered by NTSR2 is cell-dependent; after ligand activation, IP3 induction is followed by Ca^2+^ mobilization, arachidonic acid release and stimulation of MAPKs activity [58]. Differential results are reported between species and cell types, but in general, SR48692 functions as an NTSR2 agonist and SR142948A as an antagonist, while levocabastine shows both effects depending on the administered dose [51, 59-61]. The NT analogues reported in this review are listed in Table **1**. Like NTSR1, NTSR2 is internalized after NT binding, triggering signaling by ERK1/2 [61-63]. Transfection of murine NTSR2 into human cell lines, has shown that NTSR2 can be recycled to the plasma membrane using the neuron-enriched endosomal protein of 21 kDa (NEEP-21) chaperone protein [64]. Recycling of the murine NTSR2 depends on the phosphorylation of a single tyrosine residue (Tyr237), located in the intracellular loop I3. In humans, Tyr237 is replaced by Cys237 and the receptor is not recycled [65].

### 2.3 Neurotensin Receptor 3 (NTSR3)

NTSR3 is the third discovered NTSR, the first isolated in the human brain [66]. It is also called Sortilin1, due to its relation with a sorting lysosomal receptor that mediates transport from Golgi to vacuole, initially identified in yeast [67]. NTSR3 belongs to the Vacuolar protein sorting 10 protein (Vps10p) transporters family. These sorting receptors have been found to be essential for maintaining neuronal survival as they control the intracellular trafficking of target proteins [68]. Unlike the other two NTSRs, NTSR3 is a type I receptor of 100 kDa with a single TMD, which is identical in humans and mice [2].

In the brain, NTSR3 is primarily found in sensory systems and motor control areas, such as the frontal cortex and the subthalamic nucleus [49]. It is also located in oligodendrocytes [69] and in microglia. Microglial cells express only NTSR3 [70, 71]. It can mediate migration and proliferation processes [72, 73] and inflammatory responses *via* the MAPK and IP3/Akt pathways [70, 73, 74]. NTSR3 is also expressed in the spinal cord, skeletal muscles, heart, thyroid, placenta, and testes [75] and in adipocytes [76].

In neurons, NTSR3 is localized in neuronal cell bodies and dendrites [77], a subcellular localization reflecting its primary function of trafficking. NTSR3 undertakes the transport and secretion of proteins from the endoplasmic reticulum (ER) to the extracellular matrix or in the trans-Golgi network [78]. Only 5-10% of the receptor is membranous, whereas most of the receptor is located in the cytoplasm, interacting with the Golgi apparatus, vesicular organelles, and ER saccules (2,5,9). There, it is involved in protein transport from the Golgi to the plasma membrane, secretion within exosomes or secretory vesicles or the sorting to undergo lysosomal-mediated degradation. It also plays a pivotal role in the regulation of neurotrophic and neurotensinergic signals. NTSR3 interacts with the neurotrophin receptor p75^NTR^, and acts as a co-receptor for binding of pro-neurotrophins (pro-NTs), inducing cell apoptosis, with data supporting that pro-NTs can induce neuronal cell death *via* the activation of the NTSR3/p75^NTR^ complex [79, 80]. Subsidiary to this role, NTSR3 can link mature and immature NTs and transport them from the Golgi to the membrane, from where they are secreted [81]. NTSR3 has been implicated in diabetes and cardiovascular diseases [82, 83].

## 3 THE NEUROTENSINERGIC SYSTEM IN STRESS AND STRESS-RELATED DISORDERS

Stress is a state of emotional or physical tension, which can occur after traumatic events during an individual’s lifetime. Stress can be acute and/or chronic, and responses to stressful stimuli involve all neural, endocrine, and immune regulatory systems [84]. In a stressful environment or state, a certain individual can be prone to the development of depressive, anxiety, and traumatic stress disorders [85]. Anxiety is increasingly common and appears to have become more widespread over the last five years. This is partly attributed to global events such as pandemic, wars and natural disasters. According to recent reports, the COVID-19 pandemic has conferred a 25% increase in the prevalence of anxiety and depression worldwide [86]. Stress is also involved in the pathophysiology of a plethora of additional diseases. Primarily, these disorders include post-traumatic stress disorder (PTSD), major depressive disorder (MDD), and anxiety disorders [85]. However, many other health problems can originate from chronically developing stress, including comorbid mental health conditions, such as headaches and substance abuse [87], as well as somatic outcomes, including high blood pressure, cancer, cardiovascular diseases, diabetes, gastrointestinal disorders with primarily obesity, and skin problems, such as acne or eczema [87, 88] and, importantly, neurodegenerative diseases, such as Alzheimer’s disease [89, 90], Parkinson’s disease [91], schizophrenia [92] and epilepsy [93]. Here, we focus on data indicating the involvement of the NT system in anxiogenic pathways and stress-related disorders that have shown to have some implications to its neuroendocrine regulation.

Evidence that NT and NTSRs are involved in depressive and anxiety-related situations first come from their brain expression and distribution. NT, NTSR1, and NTSR2, are present in several brain regions that are implicated in anxiety and depression, such as the prefrontal cortex, amygdala, and hippocampus [53, 54, 94, 95]. The bulk of the data pointing to NT’s contribution into (patho)physiological routes derives from studies employing animal models representing anxiety and depressive responses. Due to the high complexity between physiological stress-response circuits, anxiety disorders, and the comorbidity with MDD, any such animal model must account for the considerable biological response and symptom overlap between them and related findings are to be considered in this light [96].

The role of NT in stress disorders has been hinted by early reports. Recently, NT was shown to play a role in chronic unpredictable stress rats, a preclinical rat model of chronic stress-induced anxiety-like behaviors, that involve all PTSD, MDD, and anxiety [97]. In these rats, NT was examined in the bed nucleus of the stria terminalis area, whose activation triggers anxiety-like behaviors, and as such, it contributes to chronic stress. Also, many neuropeptides are present there in high concentrations. By using brain slice neurophysiology, it was found that in chronic unpredictable stress rats, in all recorded neurons, post-synaptic depolarization induced the release of vesicular NT, resulting in an increase in the nucleus inhibitory synaptic transmission. In contrast, in non-stressed rats, only half of the recorded neurons were depolarized. As a result, these rats macroscopically showed a strong avoidance of open spaces, indicative of chronic stress. *NTSR1* mRNA was decreased in chronic unpredictable stress rats, in contrast to *NT* and *NTSR2* mRNAs that remained unchanged, possibly as a compensatory mechanism to reduce increased NT activity [97].

### 3.1 Stress Responses and Anxiety

Several initial studies converged on the active role of ΝΤ in stress and its manifestations. In rats, intense stress produced by cold-water swimming, increased the levels of *NT* mRNA in neurons of the lateral hypothalamus (LH), the main center of action of NT in the brain [98], while the non-receptor type-specific NTSR antagonist SR142948A inhibited the stress-induced increase in thermal hypersensitivity measured by the paw withdrawal latency [99]. NT microinjection into the ventral pallidum induced an anxiolytic effect in male Wistar rats, shown by its ability to increase the time spent in open elevated plus maze arms and in the center of an open field. Pretreatment of rats with the NTSR1 antagonist SR48692 abolished the effects of NT, without influencing locomotor activity [100]. Recently, increased anxiety-like behaviors, such as diminished exploration and passive stress coping strategies, were correlated with increased NT-like immunoreactivity in the cingulate cortex and periaqueductal grey matter of high anxiety-related behavior (HAB) male rats and in the hypothalamus of HAB female rats, compared to low anxiety-related behavior (LAB) rats [101].

Furthermore, acute stress in mice, reflected by their psychological stress, induced by a novel environment when transferred to a new cage, was reduced by the chemoinhibition of LH NT neurons. These mice had a faster sleep onset and reduced arousal compared to control mice. In the case of metabolic stress induced by fasting, physiological stress, wakefulness and locomotor activity were increased compared to controls [102]. These effects were opposite to the mice's normal reactions, as normally, during prolonged food absence, wakefulness, and locomotor activity decrease, so to maintain energy. Thus, NT neurons may be vital for integrating and mediating responses to stress.

More recently, NT expressing neurons in the lateral septum (LS), were found to be acutely activated by restraint stress and evoked escape behavior. *NT* as well as activity-dependent gene *Fos* mRNA were colocalized in these neurons, thus showing that both NT neuronal activity and NT expression in the LS increase during the escape action. These LS NT neurons also respond to stress when flight is a viable option. They were not active during the immobilization phase, but only before or after immobilization when they can struggle, indicating that these neurons are selectively tuned to flight responses [103]. NT, as well as NTSR1 agonist PD149163 and NTSR2 agonist JMV-431, all reduced stress-induced 22-kHz ultrasonic vocalizations after electric footshock in rats [104]. This anxiolytic effect occurs likely through the alteration of glutamatergic neurotransmission in the amygdala area, a key area of fear conditioning processes, containing dense NT fiber terminals [77]. NT neurons in several locations, such as the median raphe nucleus, become activated by stress, and function to attenuate the stress-induced response of serotonergic neurons in that area. SR48692, known to be an NTSR1 antagonist as well as an NTSR2 agonist, augments sound stress-induced activation of serotonergic neurons in that area that is blocked following administration of exogenous NT [105].

NTSR1 KO mice show increased anxious behaviors, as demonstrated in the open field activity test. These mice traveled less distance and spent less time in the center and more time in the corners of the field, as compared to wild types [106]. These anxiety-like behaviors following NTSR1 KO were also shown by Prus *et al.* [107], using the open field as well as the tail suspension test. The NTSR1 agonist PD149163 significantly reduced stress-induced 22-kHz vocalizations in rats, and conferred anxiolytic effects after footshock [107]. The mouse defense test battery is a model useful for assessing many aspects of defensive behaviors, including flight, risk assessment, defensive threat, and attack behaviors, and thus resembles emotional and anxiety disorders. This model consists of directly confronting the threat, *i.e*., forced contact with a larger animal-rat -with the option to escape or not-, measuring the responses, *i.e*., bites, and emotional challenge with the presence of another dead animal in the same environment, *etc*. SR48692 significantly modified the risk assessment or flight, similar to the benzodiazepine diazepam, and in contrast to other neuropeptide receptor antagonists, that had no effect [108]. SR48692 decreased flight reactions in the rat avoidance test after the rat was introduced into the apparatus, and decreased mice’s defensive threat and attack reactions escape attempts. Even though the antagonist showed efficacy on only some, and not all, anxiety-related responses, the authors claimed that NTSR1 blockade can play a role in the adaptative responses to unavoidable or extreme stress events, and could be useful in affective disorder and anticipatory anxiety. In the same line, NTSR1 agonist PD149163 infusion in the prelimbic cortex produced anxiogenic effects, while NTSR1 antagonist SR48692 and *NTSR1* downregulation in the prelimbic cortex by shRNA attenuated anxiogenic effects in restrained stressed rats. The NTSR2 antagonist NTRC824 had no effect on anxiogenic responses [109]. In addition, in the *NTSR1* gene, three specific single nucleotide polymorphisms (SNPs) are involved in the biological mechanisms of intermediate defense mechanisms, as found by genotyping of Han Chinese males but not females [110]. These results illustrate the role of NTSR1 in mediating anxiety and defense related processes and highlight a potential intervention target.

NTSR2 seems also to be involved in stress. The release of endogenous NT in response to stress requires the presence of NTSR2 to stimulate corticotropin-releasing factor (CRF)-induced elevation of plasma corticosterone [99]. The NTSR2 agonist β-lactotensin, when administered either intraperitoneally (i.p.) or orally, demonstrates anxiolytic-like activity. β-lactotensin injected mice increase the time mice spent in open elevated plus maze arms and in the center of an open field, and show an increase of glial cells fluorescence activity of intracellular Ca^2+^ flux [111]. These effects were not observed in NTSR2 KO mice, confirming the role of this receptor type [111].

NTSR3 protein expression is upregulated in the neocortex and hippocampus of chronically stressed mice and has been associated with decreased mobility in the open field test, while NTSR3 deficient mice displayed increased anxiety-like behavior in the open field and elevated plus maze test [112]. These results highlight the involvement of this receptor type in mediating chronic stress consolidation.

Recently, a comparison between 20 healthy individuals and 60 male and female patients with acne vulgaris (AV), revealed higher NT serum levels in the latter [113]. AV is a skin compromising and esthetic problem, to which pathogenesis and maintenance stress contributes greatly, and has a great impact on the quality of life. NT was positively correlated in AV patients with stress, depression, and anxiety. Furthermore, NT levels were significantly elevated in patients with severe acne, post-acne scars and hyperpigmentation, thus proposing NT as an objective mirror to follow acne and its psychological impairment [113]. In line with these data, *NT* mRNA and serum levels were higher in summer versus autumn in bovine endometrium, markedly influenced by heat stress [114]. These data have led to considering NT in many cases as a biochemical marker of stress response in certain manipulations [115].

### 3.2 Major Depressive Disorder (MDD)

NT-like increased immunoreactivity has been documented in a “depressed” rat strain, the Fawn-Hooded rats, which is characterized by behavioral patterns that are analogous to depression and anxiety aspects in humans. Namely, they show increased immobility in the forced swim test, and have a dysfunctional serotonergic system, with altered behavioral responses to serotonin agonists [116]. In these rats, NT was higher in the occipital cortex, frontal cortex, and hypothalamus areas. Moreover, following electroconvulsive treatment in these depressed rats, NT was reduced, indicating a correlation between this neuropeptide and depression, as well as the response to treatment. NT levels also differ following maternal separation in a rat genetic model of depression, the Flinders sensitive rats, compared to non-depressed resistant rats. NT-like immunoreactivity was increased in the nucleus accumbens, hippocampus and entorhinal cortex, and decreased in the amygdala [117].

The effects of NT on locomotor activity and its locomotor-independent antidepressant effects were also studied in corresponding tests in rats [118]. A 5 μg NT dose in the ventral tegmental area (VTA) increased the locomotor activity by two hours. Lower doses had no effect on locomotion. However, lower doses (0.5 and 1 μg) significantly increased the time the animals spent swimming -indicative of struggling- in the forced swimming test, with no changes in their general motor activity, an effect also induced by amphetamine and desipramine, psychostimulants with known anti-depressive action, in the same test. This effect was blocked by sulpiride, a specific DA receptor blocker [118]. In a more recent report, it was shown that activation of VTA NTSR1 neurons led to weight loss in obese mice, while it suppressed the effort of animals to work in order to obtain palatable rewards, such as sucrose, even when there was high motivation to consume [119]. Both the aforementioned studies in VTA neurons concluded that the antidepressant-like effects of NT are exerted by increased DA transmission, upon an NT-DA system interaction. This is supported by the fact that DA and its receptors, as well as NT [120] and NTSR1 and NTSR2 [56], are expressed in VTA neurons.

Furthermore, directly linked to post anxiety-related symptoms, LH NT neurons can effectively restrain motivated feeding in hungry, food-deprived mice. Thus, enhanced NT action could be useful to suppress the increased appetitive drive that occurs following weight loss and to prevent weight regain [121]. In the same line, mice on a high-fat diet treated with a monoclonal antibody against NT, could lose weight significantly faster than the control PBS-treated group, possibly as a result of reduced stress. Obese mice under treatment were less depressed, indicated by their fight for survival in the forced swimming test, and less anxious, spending more time in the lighted area in the light/dark box test [24]. A clinical study pointed out that NT levels in female obese patients were positively correlated with perceived stress, anxiety, depressiveness, and eating disorder symptoms, which could be influenced by a higher prevalence of mental disorders in women and by sex hormones [122].

NTSR1 KO mice show despair behaviors in the tail suspension test, as they exhibit a higher number of bouts of immobility on the second day [106]. These mice had an affected sleep pattern, with a lower percentage of sleep time spent in rapid eye movement (REM) sleep in the dark phase, and higher diurnal variation in REM sleep duration than wild types. Following sleep deprivation, NTSR1 KO exhibited more wake and less non-REM rebound sleep. Since anxiety and depression are closely linked with disturbances in sleep, the neurotensinergic system may contribute to a common shared pathway involved in both sleep and affective disorders, which includes NTSR1 [106].

All the more, the NT system has been studied in the fear alteration process in rats stressed by maternal separation [94], a major stress event that can lead to depression. In the adult rat amygdala, freezing symptoms, arising by fear-conditioned stress following maternal separation, resulted in a reduction of *NTSR1* gene expression, but not of *NT* nor *NTSR2*. Intracerebral (i.c.) microinjection of the NTSR1 antagonist SR48692 increased the percentage of freezing in a conditioned fear, whereas injection of the NTSR1 agonist PD149163 decreased freezing. This study also examined DNA methylation in the promoter region of *NTSR1* in the amygdala, and it was found to be increased after maternal separation [94]. This epigenetic modification can lead to decreased receptor expression, contributing to the development of anxiety disorders and depression in adulthood *via* a more complex gene-environment interaction.

Depression occurring after stroke, or post-stroke depression (PSD), was also shown to be attenuated by an NTSR1 agonist. Following a focal ischemic stroke in mice, depression and anxiety-like behaviors arise 4-8 weeks after the stroke. In a relevant model, treatment for 6 h with the hypothermia-inducing NTSR1 agonist HPI-363 45 min following stroke prevented long-term depression and anxiety-like behaviors examined at 6 weeks post-stroke. The agonist-treated stroke mice showed increased sucrose consumption in the sucrose preference test, used to evaluate anhedonic behavior, reduced immobile durations in the tail suspension and forced swim tests, and increased traveling distance in the central region in the open field test [123]. A previous study reported an increase in immobility behavior in NTSR1 KO mice [124]. Thereupon, NTSR1 agonist PD149163 significantly reduced time spent immobile in the forced swim test, exhibiting an antidepressant-like effect, similar to the antidepressant imipramine [125].

The role of NTSR3 in MDD is examined in several studies [126]. In the chronic unpredictable mild stress mouse model of depression, NTSR3 was significantly increased in the prefrontal cortex and hippocampus in depressed mice compared to control mice. NTSR3 overexpression in either the prefrontal cortex or hippocampus induced depressive-like behaviors, and these behaviors were alleviated by both NTSR3 knockdown and SR33557, an inhibitor of NTSR3 downstream byproduct [127]. Also, NTSR3 upregulation in mice's neocortex and hippocampus is associated with increased depression-like behavior in the forced swimming test [128]. In addition, elevated hippocampal NTSR3 protein levels in C57 mice with chronic unpredictable mild stress decreased upon treatment with fluoxetine and clozapine. In these depressed animals, imbalance occurs in the proBDNF/p75^NTR^/NTSR3 signaling pathway, with the authors claiming that intervening in this balance might provide a novel therapeutic strategy [112].

Previous reports did not show NT alterations in human patients with major depression, when measuring NT post-mortem levels in the amygdala, although MDD patients' NT levels tended to be lower than controls [129]. Other studies measured NT concentrations in cerebrospinal fluid (CSF) in healthy individuals and in depressed and schizophrenic patients. CSF NT concentrations were found significantly lower in schizophrenic patients and lower (not significantly) in depressed patients [130]. However, upon measurements of NT plasma levels in 37 depressed patients of both sexes hospitalized for severe suicidal ideation or a recent suicide attempt, the number of lifetime suicide attempts was positively correlated with NT, although the risk for suicidal behavior was attributed to the increase in pain threshold, where NT is also implicated [131]. Moreover, the *NT* gene was found to harbor deleterious mutations for depression by whole genome sequencing in African Americans [132]. Finally, serum NTSR3 levels were significantly increased in 152 depressed patients as compared with 216 healthy individuals [133]. The NTSR3-derived propeptide, an NTSR3 byproduct, was measured in the serum of 45 patients with treatment-resistant depression, who underwent electroconvulsive therapy, that has been widely used as a therapy for MDD during the past decades [134]. This peptide was found significantly increased in patients who responded to electroconvulsive therapy, showing anti-depressant effects, but not in the non-responders. Thus, the authors claimed that NTSR3-derived propeptide could contribute to the evaluation of therapy success.

### 3.3 Posttraumatic Stress Disorder (PTSD), Valence, Fear Memory

PTSD is a stress and depression-related disorder. Exposure to extreme traumatic events, such as natural disasters, and accidental and other man-made traumatic events, can lead to its occurrence. It encompasses manifestations such as reexperiencing of the trauma, numbing of responsiveness, avoidance, and hyperarousal [135]. Pathophysiological pathways involve hypothalamic-pituitary-adrenal (HPA) axis hyperactivation [136], while several direct and indirect evidence implicate the neurotensinergic system. *NTSR2* has been filtered out as an important gene for PTSD susceptibility in a cohort-based study of 63 PTSD versus 62 control samples from the NCBI GEO database among 57 other genes [137]. In the PTSD mouse model of repeated unpredictable stress, showing hippocampal abnormalities and dysregulation in amygdaloid neurotrophic and glutamatergic signaling, NT protein levels increased in the hypothalamus, but were not significantly increased in comparison to control mice [138]. NT acts primarily in the hypothalamus by inducing hypothermia following acute neuronal damage. Interestingly, patients with PTSD and concurrent major depression, showed high serum levels of the degradation enzyme of NT, prolyl endopeptidase, as compared to healthy individuals [139]. Thus, increased degradation of NT may be related to PTSD.

Appetitive or defensive behaviors can drive valence, *i.e*., the classification of memories as positive or negative and their imprint as such [140]. Traumatic experiences not only leave an indelible impression on the brain in the form of PTSD, but imaging and imprinting of rewarding or aversive behaviors can predict the future severity of PTSD [141]. The neurotensinergic system regulates valence, as shown by its actions in several brain regions [140, 142, 143], through which it can therefore be implicated in the pathogenesis of PTSD. By optogenetic stimulation using Cre transgenic mice, the effects of NT-positive neuronal subpopulations were examined for their impact on valence behaviors. Freezing response was tested by exposing NT-Cre mice to a 20 Hz blue light pulse, showing that stimulation of central amygdala (CeA) NT neurons did not elicit freezing. Following optical intracranial self-stimulation, commonly used for evaluating brain reward behaviors, activation of NT neurons resulted in self-stimulation. These observations combined show that NT neurons drive appetitive but not defensive behaviors [140]. In the same line, NT neurons in IRES-Cre mice were found to convey positive valence [135]. In the real-time place preference test, stimulation of CeA NT neurons projecting to the parabrachial nucleus, that regulates drinking and feeding behaviors, resulted in a significant place preference outcome, compared to unstimulated rats [142]. In a recent key study, NT was found to mediate valence in the basolateral amygdala (BLA) region. By CRISPR gene editing approaches, positive valence assignment was flawed as a result of NT absence of signaling in the BLA., while negative valence was not blocked but enhanced [143]. Other reports independently confirmed this NT-induced positive reinforcing effect. NT microinjection in the CeA [144], ventral midbrain [145], or ventral pallidum [146] induced conditioned place preference in adult male rats, while pretreatment with NTSR1 antagonist SR48692 abolished positive reinforcement in all areas in those distinct studies. Since emotional valence can effectuate conditions such as PTSD, further unraveling these neuromodulatory circuits could strengthen the idea of using NT and NTSRs as potential targets for therapeutic interventions.

Common symptoms that often arise with PTSD are fear expression, and substance or drug abuse [147]. Both stress and fear circuit interactions are highly implicated in the development, maintenance, and treatment of PTSD [148]. The neurotensinergic system has been studied thoroughly in several aspects of these conditions. NT expression has been documented in neuronal CeA subpopulations that control fear circuitries, by ISH [149]. Regarding fear shock, systemic administration of the NTSR1 specific agonist PD149163 produced anxiolytic-like effects in a dose-dependent manner, and attenuated fear-potentiated startle in rats, whose fear of a light flash they had associated with a footshock was reduced [150]. Yamada *et al.* [151] induced fear conditioning in adult NTSR1 KO and wild type mice, measuring their freezing responses at the exposure to evaluate fear memory. By one week of unconditioned footshock fear stimulus, NTSR1 KO mice showed a higher freezing rate than wild type mice, that disappeared when a more intense stimulus was applied, leading to the conclusion that NTSR1 mediates contextual fear memory [141].

The role of NTSR2 has been studied more in fear memory and acute stress, and the cells that express NTSR2 can modulate fear memory in the mouse brain. Acute restraint stress in mice leads to the lower number of head-dips and increased head-dip latency in the hole-board test. Both NTSR2 and an NTSR2 agonist, the peptide β-lactotensin, have been shown to weaken fear conditioning and attenuate the effects of restraint stress in mice [152, 153]. Prior administration of β-lactotensin to restrained animals ameliorated the stress-related behaviors, reducing the duration of freezing responses caused by fear conditioning [152]. These anti-stress effects of β-lactotensin were abolished by levocabastine, which was here used as an NTSR2 antagonist. NTSR2-deficient mice were subjected to contextual fear-conditioning tests, showing lower freezing responses compared to wild-type mice, both at early and late stages after the footshock and without alteration of their locomotor activity. Moreover, *NTSR2* mRNA expression was detected in the pyramidal cell layer, and in the oriens and radiatum layers of the hippocampus, as well in the hypothalamus and globus pallidus, in mice brains, all key areas of cognitive and memory functions, and in astrocytes [153]. In a more recent study, *NTSR2* mRNA was found to be strongly expressed in fear-inhibiting (or “fear-off”) neurons in the BLA, *i.e*., specific neuronal populations that are active during the expression of fear extinction [154]. During extinction retention in fear-conditioned mice, NTSR2 agonist β-lactotensin decreased freezing responses significantly, thereby enhancing fear extinction consolidation. This behavior resulted from the activation of NTSR2 in the BLA, as shown by halorhodopsin expression [154].

In a dental research study of 2022 [155], the *NTSR1* gene was found to relate to another form of fear, dental care-related fear and anxiety (DFA), which is linked to poor oral health and decreased quality of life. The SNP rs8124973 located inside the *NTSR1* coding region was identified by a genome-wide association study in 4 patient cohorts from the US and UK, to associate with a subscale of DFA, specifically on the avoidance of treatment, in the meta-analysis that was performed, following DFA survey assessment and genotyping [155]. Thus, this kind of fear, like others, also consists of a genetic component, where causal variants can play an important role.

### 3.4 Ethanol Consumption

Alcohol/ethanol consumption is a common symptom that appears after stress and depression, and more studies postulate that prolonged and excessive alcohol consumption is a potent stressor, evoking persistent dysregulation of brain reward and stress systems beyond normal homeostatic limits [156]. NT’s role in ethanol usage began from studies in laboratory mice strains, referred to as “long-sleep” (LS) and “short-sleep” (SS) [157], used to test acute alcohol sensitivity [158]. NT markedly enhanced ethanol sensitivity, assessed by ethanol-induced anesthesia and increased sleep duration, two-fold in rats [159], and up to three-fold in mice, in a dose-dependent manner [160]. The role of the neurotensinergic system in ethanol consumption was launched and consolidated by Gene Erwin and his colleagues [161-172]. Firstly, they showed that this NT-induced enhancement of ethanol sensitivity was different in SS compared to LS mice. At commonly administered NT doses of 0.5-10 μg i.c.v., NT enhanced the sensitivity of both SS and LS mice to ethanol-induced anesthesia. Ηowever, at lower doses of 5-500 ng, NT increased ethanol sensitivity in SS, but not in LS mice, as measured by the duration of loss of righting response or by respective blood ethanol levels [161]. In the hypothalamus, the primary area of NT-induced actions, NT endogenous expression, measured by its immunoreactivity *via* radioimmunoassay, is 50% higher in LS compared to SS mice [162, 163]. Thus, it is highly possible that some of ethanol’s actions are mediated by the neurotensinergic system. In the same direction, acute administration of ethanol *in vivo* produced a rapid and of long duration dose-dependent decrease of NT immunoreactivity in several brain regions, including the hypothalamus, which was abolished as the dose increased, in both SS and LS mice, and which was independent of the blood ethanol levels [163]. Presumably, this NT decrease reflects an ethanol-stimulated release, with a subsequent rapid NT degradation by endopeptidases, similar to DA ethanol-followed release in the nucleus accumbens [164]. Conversely, chronic ethanol treatment with low doses, resulted in an increase in NT immunoreactivity levels, up to 100%, in the nucleus accumbens and caudate putamen [165]. Significantly lower concentrations of NT were found in the frontal cortex of alcohol-preferring rats, in comparison to non-preferring or naïve rats [166]. Moreover, NT69L, an NT analogue with higher brain penetration and resistance to degradation than NT, that binds to both NTSR1 and NTSR2, markedly reduces ethanol preference and consumption in mice [167].

In parallel, Erwin and colleagues found that both high and low affinity NTSRs are more evident in SS relative to LS mice, as shown in binding assays in the nucleus accumbens, hippocampus, ventral midbrain, and entorhinal cortex [168]. Therefore, the changes in NTSRs affinity and density were evaluated after acute and chronic ethanol treatment [169, 170]. Both the high and low affinity subtypes were down-regulated, and high-affinity sites were affected more in certain brain regions, such as the entorhinal cortex and the nucleus accumbens. Interestingly, the maximum effect on NTSRs was seen after 2 weeks of chronic ethanol consumption, when the maximum tolerance for ethanol has been developed in the animals, and NTSRs properties returned to control values 3 weeks after withdrawal from ethanol, concomitantly to the return to control behavioral responses to ethanol [169]. Based on these data, quantitative trait loci (QTL) were explored, correlating simple sequence length polymorphisms with NTSRs densities and NT brain immunoreactivity, combined with the effects of ethanol in SS and LS mice. Initially, a QTL found in chromosome 2, where NTSR1 is expressed, was linked to the *NTSR1* gene, and was correlated with the sedative effects of ethanol [171]. Subsequently, other QTLs were revealed, with a significant correlation between NTSR2 density in the frontal cortex and voluntary ethanol consumption in the above-mentioned mice [172]. Taken together, these data underscore NT signaling may regulate ethanol consumption behaviors.

The CeA plays an important role in alcohol use and other affective disorders. CeA NT neurons in IRES (internal ribosome entry site)-Cre mice, were activated following voluntary consumption of relatively low ethanol doses, but not of sucrose or saccharin (both sweet fluids), nor quinine (intense bitter fluid), as shown by an increase of the expression of the activity-dependent gene *Fos* [142]. Selective genetic ablation of CeA NT neurons led to a decrease in ethanol consumption compared to water or other fluids, in the two-bottle choice, where mice chose freely between a bottle containing ethanol and one containing water or other fluid, with the positions of the bottles changed continuously to control for position preferences. The same decrease in ethanol consumption was also observed during another drinking preference assay, this time with a longer exposure and higher dose of alcohol, in the course of intermittent access test, in which animals are given access to both a bottle of ethanol and water for half a week on consecutive days, and two same bottles of water on the other days. Photostimulation of CeA NT neurons projecting to the parabrachial nucleus, an area with a characterized role in drinking and feeding behaviors, led to an increase in consumption of sweet fluids, along with ethanol consumption, but not of quinine or water, in the NT-Cre mice [142]. Thus, NT seems to regulate ethanol-specific voluntary consumption in several brain areas in nonalcohol-dependent mice, and NT neurons are activated by, and promote, ethanol consumption.

The role of both NTSR1 and NTSR2 in the above-mentioned processes has been studied by evaluating the effects of the elimination of each respective receptor. NTSR1 KO led to increased ethanol consumption and preference in mice compared to wild type animals, assessed by a two-bottle choice drinking experiment. Injection of the NT analogue NT69L for 4 days, resulted in reduced consumption and preference for ethanol, and increased ethanol sensitivity and thus intoxication, in wild type, but not in NTSR1 KO mice [167]. NTSR2 KO also led to increased ethanol consumption and preference in mice, compared to wild type animals. These NTSR2 KO mice did not show any preference for nor quinine, showing that their taste is similar, but neither for saccharin; thus, the elimination of NTSR2 may also implicate in the reward behavior [173]. Upon injection of NT69L, consumption and preference for ethanol were reduced, both in wild type and NTSR2 KO mice, however, both were lower in NTSR2 KO mice. Also, both consumption and preference in the NTSR2 KO mice were more reduced at post-injection, rather than pre-injection, compared to wild type mice. The effect of NT69L on ethanol preference was not fully recovered after the injection period, unlike for consumption [173]. Therefore, NTSR1 may regulate more the intoxication effect of ethanol, and NTSR2 the hedonic feeling of consumption, while both receptors could be implicated in ethanol intake. This has been further suggested recently by NTSR2 regulating chronic excessive ethanol drinking, as chronic high drinking rats had higher NTSR2 protein levels in the paraventricular thalamus. When NTSR2 was stimulated in that area by the NTSR2 agonist JMV-431 in low drinking rats, it induced their exploratory behavior that predicted the amount of subsequent ethanol drinking, ultimately resulting in low drinking animals to resemble the behavior of high drinking animals [174]. Association between two SNPs in the *NTSR1* gene and alcohol dependence in the Han Chinese population regarding allele and genotype frequencies, have been reported [175].

It is possible that upon changes in one receptor, the other could take over its function in a specific setting. In COS-7 cells, NTSR2 induces intracellular sequestration of NTSR1, thereby regulating NTSR1 activity and its signaling. In fact, heterodimerization of NTSR1 and NTSR2, can decrease the cell surface density of NTSR1 [176]. Another study showed that NTSR1 null and NTSR2 null mice, exhibit reciprocal upregulation of *NTSR1* and *NTSR2* mRNA expression, compared to wild-type mice, in the entire brain [177]. Thus, the upregulation of NTSR1, which already mediates some of ethanol’s actions, may explain the normal level of sensitivity to ethanol-induced ataxia and locomotor suppression, exhibited by NTSR2 KO animals [173].

Regarding *NTSR3*, its mRNA levels, as well as those of p75^NTR^, were found to be increased in a clinical study in the peripheral blood of 30 male patients with alcohol dependence, compared to healthy controls, while NTSR3 protein levels were similar to controls, despite p75^NTR^ increase. Both NTSR3 and p75^NTR^ were positively correlated with the average daily alcohol consumption. Furthermore, the proBDNF/ p75^NTR^/NTSR3 pathway is activated in alcoholic patients, also suggesting that NTSR3 may be implicated in alcohol consumption [178]. In another study of 152 depressed and 216 control individuals, the mean NTSR3 serum concentration was positively correlated with a high alcohol intake in depressed male and female individuals compared to controls, although long-term alcohol administration had no effects on NTSR3 protein levels in the mouse hippocampus [133]. Concurrently, in 30 male patients suffering from alcohol dependence, NTSR3 serum levels were found to be reduced one week after alcohol withdrawal and detoxification [179], further implicating NTSR3 in dependence.

### 3.5 Substance Abuse/Addiction

It is well-established that substance use and abuse or addiction behavior can be triggered by acute or chronic stress, and CRF and the HPA axis (CRF/HPA) are involved in these processes [180]. The neurotensinergic system has been piecemeal studied with regard to consumption and addiction for all major addictive drugs. Early on, the ability of endogenous NT on initiating amphetamine sensitization was studied [181]. The locomotor activity levels in rats treated with amphetamine alone or in parallel with the NT analogue SR48692 (NTSR1 antagonist as well as NTSR2 agonist), were the same as in controls, with attenuation of movements observed in the amphetamine alone-treated rats, as shown by the sensitization tests. SR48692 was also shown by another group to have this effect in systematically amphetamine-treated and sensitized rats, but not in rats treated acutely by a single amphetamine dose, and it reduced the locomotor activation induced by stress [182]. As such, signaling *via* NTSRs specifically affects the sensitized chronic behavioral response to amphetamine, thereby implicating drug abuse. The group of Rompré working on NT-induced amphetamine sensitization subsequently showed that an excitotoxic lesion in the prefrontal cortex attenuated the potentiation of amphetamine-induced locomotion after NT administration. The sensitization effect of amphetamine to NT administration was stronger in non-lesioned compared to lesioned rats [183]. Here, the authors claimed that this effect was due to repeated activation of NTSRs. Although in this instance, SR48692 was used as an NT antagonist, considering that it is an NTSR2 agonist, these promoting effects are possibly mediated by NTSR2.

More recently, NT and NTSRs were studied by the Beckstead group in relation to methamphetamine, a psychostimulant that exhibits significant abuse potential, leading to continuous efforts for exploitation of the neurotensinergic system for methamphetamine abuse treatment. SR142948A, the general NTSR antagonist, upon administration in VTA of mice, not only reduced methamphetamine self-administration, that is promoted by NT during initial methamphetamine exposure, but also reduced methamphetamine-seeking behavior [184]. Also, methamphetamine self-intake and active seeking were reduced in NTSR1 KO homologous and heterologous mice, and it was found that this was dependent on DA signaling in the VTA. Indeed, DA cell firing activity after methamphetamine exposure was the same in KO mice, whereas it was reduced in wild-type mice [185]. The NTSR1 agonist PD149163 was able to decrease methamphetamine self-administration in mice [186]. Along with these efforts, a small-molecule, brain penetrant NTSR1 agonist, ML314, was developed. This molecule was shown to efficiently block methamphetamine self-administration and reduce methamphetamine-associated conditioned place preference, in rats and mice, respectively [187]. SBI-553, a β-arrestin agonist, facilitates NTSR1 internalization, leading to methamphetamine self-administration reduction in mice [188].

Among others, NT and NTSRs have been studied with regard to cocaine intake and addiction. NT increased sensitization to the locomotor stimulant effect of cocaine, as ambulatory and non-ambulatory activity induced by cocaine was higher in NT-treated rats, as compared to controls [189]. During cocaine sensitization, NT KO mice showed increased locomotor stimulant cocaine-induced effects [190], while during withdrawal from cocaine, the NT analogue SR48692 (considered here as an NT antagonist) decreased locomotor sensitization and conditioned place preference in rats [191], suggesting that NT participates in the maintenance of these behaviors.

The neurotensinergic system may play a role in tolerance mechanisms to clozapine, a major antipsychotic drug. Clozapine treatment led to increased sensitivity in rats treated with the NTSR1 agonist PD149163 in three antipsychotic activity tests. This agonist induced a dose-dependent tolerance to clozapine at some of its behavioral effects [192]. The same molecule displayed antipsychotic effects in conditioned avoidance tasks. It suppressed conditioned avoidance responding, similarly to the typical antipsychotic haloperidol, without producing catalepsy, thus acting similarly to clozapine [193]. Finally, a specific *NTSR1* gene SNP, rs3915568, has been associated with vulnerability to drug addiction in general and heroin addiction in specific, in a sample of 1860 subjects of European and African ancestry [194].

## 4 CONCLUSION: NOVEL DRUG TARGETS AND HYPOTHESES

NT has been shown to antagonize anxiety and fear sentiments and promote anxiolysis and exploration behavior, acting on CRF containing neurons of the CeA, contrasting with the role of CRF itself, which promotes anxiety and fear learning [195]. The central role of CRF in stress and stress-related disorders has been studied extensively [97, 196, 197], acting mainly through activation of the HPA axis, but also as a mediator in multiple CNS and peripheral sites. NT and CRF might interact in their regulatory contribution and involvement in pathophysiological processes. Endogenous NT can elicit CRF release, as administration of the NTSR1 antagonist/NTSR2 agonist SR48692 to the PVN of the hypothalamus attenuated *CRF* mRNA and immunoreactivity and stress-induced elevations in HPA activity [198, 199]. As previously mentioned, during the conditioned place preference test in rats, NT acting in the CeA can promote a positive reinforcement or anxiolytic effect, while the molecule SR48692 blocked the anxiolytic effects of NT [144]. Pomrenze *et al.* knocked down NT as well as CRF in rats, and showed that the knockdown of CRF impairs fear learning, whereas the knockdown of NT enhances it [182]. In the same study, GABA was found to regulate baseline anxiety-like behavior. Thus, in CRF-containing neurons of the CeA, that promote anxiety-like behavior and fear learning when activated [200], NT and GABA −with the second neurotransmitter possibly evoked by the first−, can suppress anxiety and fear [195].

Although CRF does not show any efficacy in enhancing ethanol-induced anesthesia, neither in SS nor in LS mice, it is able to decrease ethanol sensitivity in LS but not in SS mice, that are more prone to ethanol, showing an early complementing pattern of action of NT and CRF [161]. Due to the series of studies mentioned above that provide evidence for a preference for ethanol in particular and not other fluids, whether palatable or not, other potential mechanisms by which chronic ethanol might alter NT binding characteristics, such as those involving the well-known membrane fluidizing effects of ethanol, fail. Therefore, further research into these mechanisms is warranted. Interestingly, common QTLs that have been identified in NTSR1 and NTSR2 genes indicate that genes regulating NT and NTSRs expression may be the same as those regulating sensitivity to ethanol.

DA is one of the most important neurotransmitters in the hippocampus. Relations between NT and DA have been well investigated. NT regulates the DA system. In particular, NT increases the firing rate of DA neurons, and has a depolarizing effect [9]. It is appealing to suggest that NT or NTSRs exert their actions by modulating DA receptors. In mesolimbic neurons, such as those of the VTA, NT and DA are co-localized and thus may be co-released [56, 163]. In the hypothalamus, a population of DA neurons express NT, and NT acts to control the mesolimbic DA system and energy balance [201].

Finally, it is most likely that some of the effects of NT and NTSRs in the aforementioned disorders, especially in relation to skin diseases and other acute stimuli, are mediated by inflammation that is exacerbated in stressful situations, possibly in cooperation also with CRF. It was first described that NT acts as a pro-inflammatory cytokine, mediating vasodilatation [202], and both NT and CRF have immunomodulatory actions [203, 204].

NT can activate mast cells after immobilization stress in rats, and SR48692 inhibits the stress-mediated secretion of mast cell products [205]. Interactions between NT-CRF augment human mast cell activation, and release of inflammatory mediators under stress [204]. This contributes to auto-immune and inflammatory diseases that corrode with stress. These are particularly relevant to PTSD, along with brain injury and other pathologies, and lead, in turn, to further NT release [206]. In mice, following the challenge with lipopolysaccharide (LPS), that stimulates inflammation and activates the HPA, treatment with the NTSR1 agonist PD149163 decreases inflammatory cytokines levels [207]. In many diseases that encompass stress, *e.g*., in autism spectrum disorders, in which patients -more commonly children- show severe anxiety, as well as skin atopic disease and eczema, both NT and CRF levels are elevated in serum [208]. In skin disorders, which are associated with increased numbers of activated mast cells and are worsened by stress, CRF induces skin vascular permeability through NT acting on mast cells, and both peptides contribute to the pathogenesis of skin disorders exacerbated by stress [209]. There appears to be a strong link between all these components, to maintain homeostasis.

Notably, we have shown a role for the neurotensinergic system in neuroinflammation, which has a direct impact on disorders such as chronic stress. We have provided evidence that NTSR2 is linked to acute neuroinflammation in epileptic rats, expressed specifically in reactive brain astrocytes and endothelial cells, and inclines in the chronic phase of neuroinflammation [53]. The role of this system in inflammatory conditions outside the CNS requires more attention.

Neuropeptide systems have been proposed as therapeutic targets for stress, depression, and anxiety disorders [210], and the neurotensinergic system may be of pivotal importance in this direction.

## CONCLUSION

In this review, we have gathered results that consolidate the role of the neurotensinergic system in stress circuits and related pathogenetic pathways, concluding that NT and its receptors play a key role in the modulation of stress-related disorders. Further studies are warranted to elucidate the implication of members of the neurotensinergic system in the brain circuits associated with different conditions. The main points of this review are summarized in Fig. (**1**).

## Figures and Tables

**Fig. (1):**
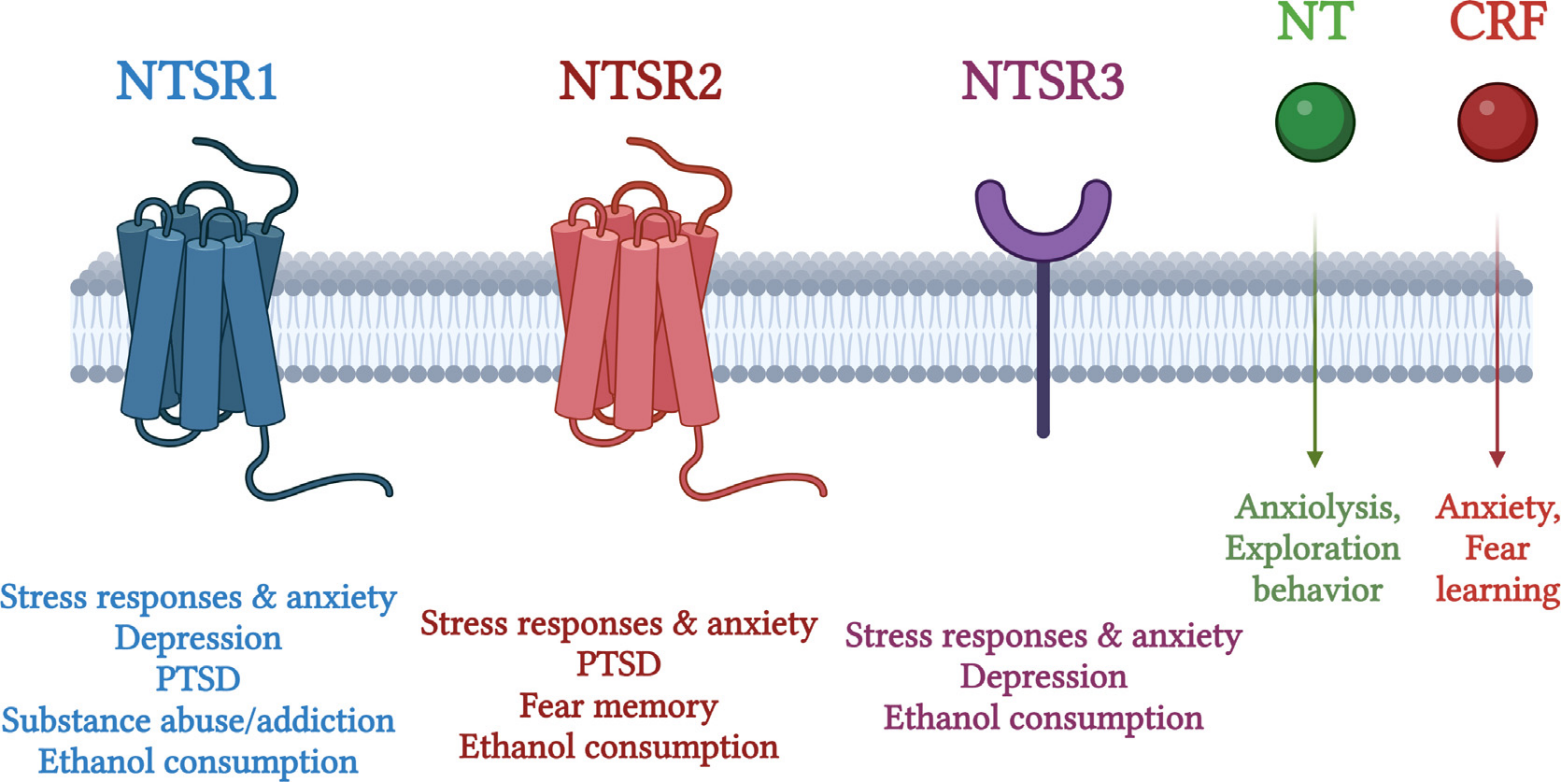
The neurotensinergic system in stress and stress-related disorders. There are three known neurotensin receptors (NTSRs), namely neurotensin receptor 1 (NTSR1), NTSR2 and NTSR3 (Sortilin1). NTSR1 and NTSR2 are G-protein coupled receptors with seven transmembrane domains. NTSR3 or gp95/Sortilin1 is a type I receptor that is not coupled to a G-protein. NTSRs mediate multiple actions and in conjunction with NT, form the neurotensinergic system. The above scheme illustrates the role and involvement of the neurotensinergic system in different aspects of the stress response and in the development of the main stress-related disorders, such as depression and anxiety, post-traumatic stress disorder (PTSD) and its associated symptoms such as fear memory and maternal separation, and ethanol addiction and substance abuse. Different receptors play distinct roles in their pathophysiology. Substance abuse/addiction involves mainly NTSR1, while fear memory involves mainly NTSR2. Stress responses and anxiety involve mainly NTSR1, as well as NTSR2 and NTSR3 in less degree. Depression involves mainly NTSR1 and NTSR3 in less degree, and PTSD involves mainly NTSR2 and NTSR1 in less degree. Interestingly, ethanol consumption involves all NTSRs. An interactive dialogue with the CRF neuroendocrine system is also hypothesized. Some of the actions of NT and NTSRs in these pathological settings may be driven through interactions between NT and CRF in their regulatory contribution, that although they exert contracting roles in stress and anxiety, they interact and impact one another’s expression.

**Table 1:** List of NT analogues and NTSRs ligands mentioned in this review and their effects in stress and stress-related disorders.

**Molecule**	**Role**	**Effect in Stress & Related Disorders**	**References**
HPI-363	NTSR1 agonist	Prevented long-term depression and anxiety-like behaviors at 6 weeks post-stroke	[123]
JMV-431	NTSR2 agonist	Anxiolysis, exploration behavior	[104, 174]
Levocabastine	NTSR2 agonist/antagonist (depending on administered dose)	Abolished anti-stress effect	[152]
ML314	NTSR1 agonist	Blocked methamphetamine self-administration, reduced methamphetamine-associated conditioned place preference	[187]
NT69L	NT analogue for NTSR1&2	Reduced ethanol preference and consumption, increased ethanol sensitivity and thus intoxication	[167, 173]
NTRC824	NTSR2 antagonist	No effect on anxiogenic responses after NTSR1 blockade	[109]
PD149163	NTSR1 agonist	Anxiolysis, the anxiogenic effect [in the prelimbic cortex], decreased freezing and fear-potentiated startle anti-depressive behavior, decreased methamphetamine self-administration, increased sensitivity after clozapine treatment, antipsychotic effects	[94, 104, 107, 109, 125, 150, 186, 192, 193]
SR142948A	general NTSR antagonist	Inhibited stress-induced increase, reduced methamphetamine self-administration, reduced methamphetamine-seeking behavior	[99, 184]
SR33557	NTSR3 downstream byproduct inhibitor	Alleviated depressive-like behaviors following NTSR3 overexpression	[128]
SR48692	NTSR1 antagonist/NTSR2 agonist (depending on cell type/species)	Abolished NT-induced anxiolytic effect, decreased flight reactions and defensive threat and attack reactions escape attempts, anxiolysis, increased freezing, abolished positive reinforcement, decreased locomotor sensitization during amphetamine treatment, decreased conditioned place preference	[94, 100, 108, 109, 144-146, 181, 182, 191]
β-lactotensin	NTSR2 agonist	Anti-stress effect, reduced fear conditioning, decreased freezing responses	[111, 152-154]

## LIST OF ABBREVIATIONS

AV: Acne Vulgaris
BBB: Blood-brain Barrier
BLA: Basolateral Amygdala
CeA: Central Amygdala
CNS: Central Nervous System
CRF: Corticotropin-releasing Factor
DA: Dopamine
ER: Endoplasmic Reticulum
GABA: Gamma-aminobutyric Acid
GPCRs: G-protein Coupled Receptors
HAB: High Anxiety-related Behavior Rats
HPA: Hypothalamic-Pituitary-adrenal Axis
i.c.v.: Intracerebroventricularly
i.p.: Intraperitoneally
KO: Knock-out
LAB: Low Anxiety-related Behavior Rats
LH: Lateral Hypothalamus
LS: Lateral Septum
LS: Long-sleep mice
MDD: Major Depressive Disorder
NT: Neurotensin
NTSR1: Neurotensin Receptor 1
NTSR2: Neurotensin Receptor 2
NTSR3: Neurotensin Receptor 3
NTSRs: Neurotensin Receptors
PTSD: Post-traumatic Stress Disorder
QTL: Quantitative Trait Loci
REM: Rapid Eye Movement Sleep
SNPs: Single Nucleotide Polymorphisms
SS: Short-sleep Mice
TMD: Transmembrane Domain
Vps10p: Vacuolar Protein Sorting 10 Protein
VTA: Ventral Tegmental Area
